# Comments on: Modified Pereira Suture as an Effective Option to Treat Postpartum Hemorrhage Due to Uterine Atony

**DOI:** 10.1055/s-0039-1681111

**Published:** 2019-03

**Authors:** Shigeki Matsubara

**Affiliations:** 1 Department of Obstetrics and Gynecology, Jichi Medical University, Shimotsuke, Japan

Authors’ Reply

Dear Editor,

Moleiro et al^1^ reported that a modified Pereira uterine compression suture (UCS) achieved hemostasis in two patients with postpartum hemorrhage. In the original Pereira suture, multiple sutures were applied both longitudinally and transversally around the uterus, with threads not penetrating the uterine wall and thus not entering the uterine lumen.^2^ ^3^ The procedure described by Moleiro et al was the combination of the B-Lynch and the Pereira sutures, and thus, they referred to it as the modified Pereira suture. I have some concerns and clarifications.

First, Moleiro et al^1^ stated that the “Pereira suture can be performed when there is no hysterotomy,” and that this is a merit of the Pereira suture. However, Moleiro et al^1^ concomitantly used the B-Lynch suture, which requires hysterotomy.^4^ Actually, the requirement of hysterotomy is considered a pitfall of the B-Lynch suture.^3^ ^5^ For the concomitant use with the Pereira suture, the Hyman suture (simple brace suture: simple B-Lynch suture)^6^ may be better, since it does not require hysterotomy.^3^ ^6^

Secondly, the rationale for the addition of the Pereira suture is unclear. Moleiro et al^1^ performed the B-Lynch suture first, followed by a transverse Pereira suture. B-Lynch or Hayman threads sometimes “slide out” laterally, failing to achieve hemostasis:^3^ ^6^ we have devised the Matsubara-Yano (MY) UCS, which prevents the threads from sliding out.^3^ ^6^ Although Moleiro et al^1^ did not mention this, the transverse Pereira suture may also prevent sliding out. On the other hand, UCSs should be simple, as long as hemostasis can be achieved.^3^ Thus, there may be 2 scenarios: 1) The Pereira suture was added since the B-Lynch suture did not achieve hemostasis, or 2) the B-Lynch suture achieved hemostasis and the Pereira suture was added prophylactically to prevent the sliding out of the thread. Scenario 1) means the emergent addition of the Pereira suture (emergent modified Pereira suture), whereas scenario 2) means the planned prophylactic addition of the Pereira suture (planned modified Pereira suture). I wish to know the fundamental concept of the modified Pereira suture.

I commend Moleiro et al^1^ for re-evaluating the Pereira UCS. To my knowledge, ∼ 30 UCS have been hitherto reported. Of them, the B-Lynch,^4^ the Hayman,^6^ the Cho,^7^ and the MY^3^ ^5^ sutures are cited in the Williams Obstetrics textbook,^8^ and these four sutures have been repeatedly reported, meaning that they were re-evaluated. However, other UCSs have been rarely re-evaluated: many researchers appear to be enthusiastic to establish new UCSs. Actually, a PubMed search retrieved only one report re-evaluating the Pereira suture.^9^ Moleiro et al^1^ have revived the Pereira suture, which should be commended. Now is the time to re-evaluate various UCSs. Such efforts may determine which UCS should be used according to individual situations.
